# Di-*tert*-butyl 3,3′-(2,2′-bi-1*H*-imidazole-1,1′-di­yl)dipropanoate

**DOI:** 10.1107/S1600536809021369

**Published:** 2009-06-13

**Authors:** Ke Wang, Yue-Jiao Li, Ting-Ting Zhang, Hong-Ze Liang

**Affiliations:** a State Key Laboratory Base of Novel Functional Materials, and Preparation Science, Faculty of Materials Science and Chemical Engineering, Ningbo University, Ningbo 315211, People’s Republic of China

## Abstract

In the title compound, C_20_H_20_N_4_O_4_, the complete molecule is generated by a crystallographic centre of symmetry. The conformation is stabilized by two intramolecular C—H⋯N links.

## Related literature

For the background to 2, 2′-biimidazole derivatives, see: Barnett *et al.* (1999 ▶, 2002 ▶); Liang *et al.* (2009 ▶); Zhang & Liang (2009 ▶); Zhang, Zhang, Ren *et al.* (2009 ▶); Zhang, Zhang, Xu *et al.* (2009 ▶). For the synthesis, see: Barnett *et al.* (1999 ▶).
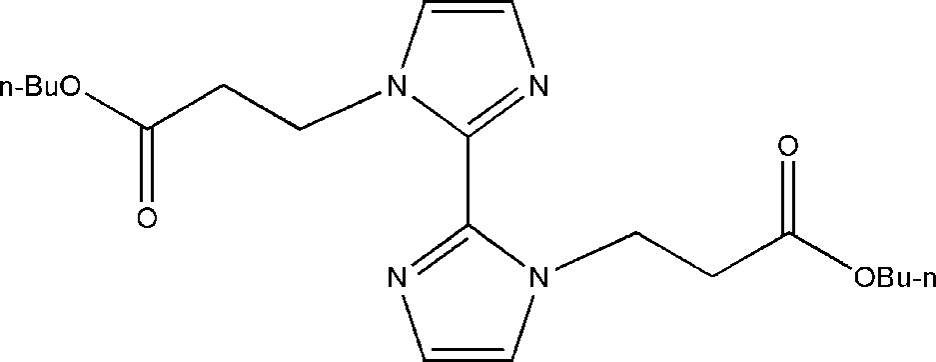


## Experimental

### 

#### Crystal data

C_20_H_30_N_4_O_4_
*M* *_r_* = 390.48
Monoclinic, 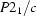

*a* = 7.0321 (14) Å
*b* = 17.484 (4) Å
*c* = 8.9681 (18) Å
β = 100.80 (3)°
*V* = 1083.1 (4) Å^3^
*Z* = 2
Mo *K*α radiation
μ = 0.08 mm^−1^
*T* = 295 K
0.48 × 0.42 × 0.14 mm

#### Data collection

Rigaku  R-AXIS RAPID diffractometer
Absorption correction: multi-scan (*ABSCOR*; Higashi, 1995 ▶) *T*_min_ = 0.960, *T*_max_ = 0.988
9620 measured reflections
2453 independent reflections
1474 reflections with *I* > 2σ(*I*)
*R*_int_ = 0.077

#### Refinement

*R*[*F* ^2^ > 2σ(*F* ^2^)] = 0.077
*wR*(*F* ^2^) = 0.190
*S* = 1.02
2453 reflections
128 parameters
H-atom parameters constrained
Δρ_max_ = 0.32 e Å^−3^
Δρ_min_ = −0.24 e Å^−3^

### 

Data collection: *RAPID-AUTO* (Rigaku, 1998 ▶); cell refinement: *RAPID-AUTO*; data reduction: *CrystalStructure* (Rigaku/MSC, 2004 ▶); program(s) used to solve structure: *SHELXS97* (Sheldrick, 2008 ▶); program(s) used to refine structure: *SHELXL97* (Sheldrick, 2008 ▶); molecular graphics: *SHELXTL* (Sheldrick, 2008 ▶); software used to prepare material for publication: *SHELXL97*.

## Supplementary Material

Crystal structure: contains datablocks global, I. DOI: 10.1107/S1600536809021369/fb2148sup1.cif

Structure factors: contains datablocks I. DOI: 10.1107/S1600536809021369/fb2148Isup2.hkl

Additional supplementary materials:  crystallographic information; 3D view; checkCIF report

## Figures and Tables

**Table 1:** Hydrogen-bond geometry (Å, °)

*D*—H⋯*A*	*D*—H	H⋯*A*	*D*⋯*A*	*D*—H⋯*A*
C10—H10*B*⋯N2^i^	0.97	2.46	2.960 (3)	111

Symmetry code: (i) 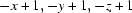.

**Fig. 1.**
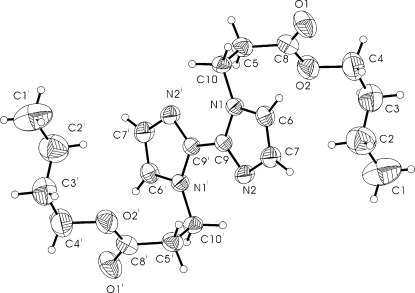
View of the title molecule with the displacement ellipsoids at the 45% probability level. The labelling of the non-H atoms is also given. The symmetry code (i):-x+1, -y+1, -z+1.

**Fig. 2.**
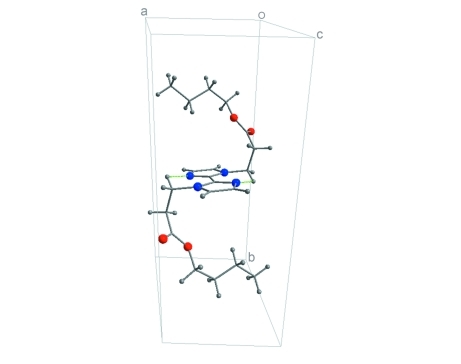
A section of the title structure. The C-H···N hydrogen bonds (Tab. 1) are shown as dashed lines.

## supplementary crystallographic information

### Comment

Biimidazole is a potentially polydentate ligand, but its chemistry is less developed in comparison to the imidazole chemistry. The reason may be a limited solubility of biimidazole in common organic solvents. Although several new disubstituted 2,2'-biimidazoles have been recently synthesized (Barnett *et al.*, 1999; Barnett *et al.*, 2002), a few metal complexes based on biimidazole derivatives are reported (Zhang, Zhang, Ren *et al.*, 2009). In the course of our ongoing study, we have successfully synthesized a series of biimidazole derivatives with terminal carboxylic, hydroxyl, phosphino, imino groups which can be used as ligands in the coordination chemistry (Zhang & Liang, 2009; Zhang, Zhang, Xu *et al.*, 2009) and cross-coupling reactions (Liang *et al.*, 2009). These ligands exhibit rich coordination patterns and catalytic properties. Here we report the synthesis and the crystal structure of the title compound which is an intermediate of those above mentioned ligands. As shown in Fig. 1, the biimidazole ring atoms (C6, C7, C9, N1, N2 and their inversion-related partners) exhibit essentially coplanar mutual orientation [the dihedral angle is 0.00 (1)°], and the value of the torsion angle C9—N1—C10—C5 is -77.53 (30)°. In the crystal structure, there are weak C-H···N interactions (Tab. 1, Fig. 2).

### Experimental

The title compound was prepared according to a published procedure (Barnett *et al.*, 1999). 0.2 g (5 mmol) of NaOH was added to a suspension of 3 g (22.4 mmol) of 2,2'-biimidazole in 100 ml of DMF (dimethylformamide) at 80°C. The resulting  mixture was stirrred for 30 min. In the course of this time the mixture gradually turned into a clear pale yellow solution. 7.12 g (55.6 mmol) butyl acrylate in 10 ml of DMF was added dropwise in several minutes to the solution and the reaction was stirred at 80°C for 8 h until the heating was stopped. The DMF was removed *via* vacuum distillation in a hot oil bath at 100°C. The resulting black brown oil was dissolved in water (30 ml) and extracted with CH_2_Cl_2_. The organic layer was washed with water, and then evaporated under reduced pressure to yield a white product (7.4 g, 85%). The product was dissolved in 95% ethanol (30 ml) and cooled slowly in a refrigerator to afford colourless block crystals of average size 1.5 mm×1.2 mm×0.5 mm that were suitable for the X-ray analysis.

### Refinement

All the hydrogens were discernible in the difference electron density map. Nevertheless, the hydrogens were situated into the idealized positions. The C-H distances were constrained to 0.93, 0.96 and 0.97 Å for aryl, methylene and methyl hydrogens, respectively. *U*_iso_(H) = 1.2 *U*_eq_(C_aryl_); *U*_iso_(H) = 1.2 *U*_eq_(C_methylene_); *U*_iso_(H) = 1.5 *U*_eq_(C_methyl_).

### Crystal data

**Table:**

C_20_H_30_N_4_O_4_	*F*(000) = 420
*M**_r_* = 390.48	*D*_x_ = 1.197 Mg m^−^^3^
Monoclinic, *P*2_1_/*c*	Mo *K*α radiation, λ = 0.71073 Å
Hall symbol: -P 2ybc	Cell parameters from 1478 reflections
*a* = 7.0321 (14) Å	θ = 3.0–27.5°
*b* = 17.484 (4) Å	µ = 0.08 mm^−^^1^
*c* = 8.9681 (18) Å	*T* = 295 K
β = 100.80 (3)°	Plate, colourless
*V* = 1083.1 (4) Å^3^	0.48 × 0.42 × 0.14 mm
*Z* = 2	

### Data collection

**Table:**

Rigaku R-AXIS RAPID diffractometer	2453 independent reflections
Radiation source: fine-focus sealed tube	1474 reflections with *I* > 2σ(*I*)
graphite	*R*_int_ = 0.077
Detector resolution: 0 pixels mm^-1^	θ_max_ = 27.5°, θ_min_ = 3.2°
ω scans	*h* = −9→9
Absorption correction: multi-scan (*ABSCOR*; Higashi, 1995)	*k* = −22→22
*T*_min_ = 0.960, *T*_max_ = 0.988	*l* = −11→10
9620 measured reflections	

### Refinement

**Table:**

Refinement on *F*^2^	Primary atom site location: structure-invariant direct methods
Least-squares matrix: full	Secondary atom site location: difference Fourier map
*R*[*F*^2^ > 2σ(*F*^2^)] = 0.077	Hydrogen site location: difference Fourier map
*wR*(*F*^2^) = 0.190	H-atom parameters constrained
*S* = 1.02	*w* = 1/[σ^2^(*F*_o_^2^) + (0.0718*P*)^2^ + 0.3943*P*] where *P* = (*F*_o_^2^ + 2*F*_c_^2^)/3
2453 reflections	(Δ/σ)_max_ < 0.001
128 parameters	Δρ_max_ = 0.32 e Å^−^^3^
0 restraints	Δρ_min_ = −0.24 e Å^−^^3^
59 constraints	

### Special details

**Table:**

Geometry. All e.s.d.'s (except the e.s.d. in the dihedral angle between two l.s. planes) are estimated using the full covariance matrix. The cell e.s.d.'s are taken into account individually in the estimation of e.s.d.'s in distances, angles and torsion angles; correlations between e.s.d.'s in cell parameters are only used when they are defined by crystal symmetry. An approximate (isotropic) treatment of cell e.s.d.'s is used for estimating e.s.d.'s involving l.s. planes.
Refinement. Refinement of *F*^2^ against ALL reflections. The weighted *R*-factor *wR* and goodness of fit *S* are based on *F*^2^, conventional *R*-factors *R* are based on *F*, with *F* set to zero for negative *F*^2^. The threshold expression of *F*^2^ > σ(*F*^2^) is used only for calculating *R*-factors(gt) *etc*. and is not relevant to the choice of reflections for refinement. *R*-factors based on *F*^2^ are statistically about twice as large as those based on *F*, and *R*- factors based on ALL data will be even larger.

### Fractional atomic coordinates and isotropic or equivalent isotropic displacement parameters (Å^2^)

**Table:**

	*x*	*y*	*z*	*U*_iso_*/*U*_eq_	
N1	0.3460 (3)	0.51573 (11)	0.30746 (19)	0.0410 (5)	
N2	0.6689 (3)	0.51901 (13)	0.3698 (2)	0.0522 (6)	
C10	0.1400 (3)	0.50398 (14)	0.3101 (3)	0.0454 (6)	
H10A	0.0628	0.5297	0.2237	0.054*	
H10B	0.1099	0.5268	0.4016	0.054*	
O2	0.2468 (3)	0.32667 (12)	0.1854 (2)	0.0717 (6)	
O1	0.0366 (3)	0.40123 (12)	0.0365 (2)	0.0774 (7)	
C9	0.5051 (3)	0.50815 (13)	0.4210 (2)	0.0415 (6)	
C8	0.1171 (4)	0.38275 (15)	0.1612 (3)	0.0525 (6)	
C7	0.6104 (4)	0.53329 (17)	0.2169 (3)	0.0568 (7)	
H7A	0.6939	0.5428	0.1501	0.068*	
C6	0.4159 (4)	0.53160 (15)	0.1774 (2)	0.0504 (6)	
H6A	0.3431	0.5396	0.0809	0.061*	
C5	0.0864 (4)	0.41992 (15)	0.3056 (3)	0.0520 (6)	
H5A	0.1644	0.3940	0.3914	0.062*	
H5B	−0.0484	0.4147	0.3144	0.062*	
C4	0.2854 (6)	0.2868 (2)	0.0492 (4)	0.0905 (11)	
H4A	0.3139	0.3238	−0.0242	0.109*	
H4B	0.1724	0.2577	0.0027	0.109*	
C3	0.4524 (6)	0.2349 (2)	0.0943 (5)	0.0958 (12)	
H3A	0.4239	0.2005	0.1722	0.115*	
H3B	0.4664	0.2039	0.0072	0.115*	
C2	0.6387 (6)	0.2728 (2)	0.1520 (5)	0.1045 (13)	
H2B	0.6290	0.3010	0.2434	0.125*	
H2C	0.6653	0.3092	0.0770	0.125*	
C1	0.8074 (6)	0.2163 (3)	0.1876 (7)	0.1289 (18)	
H1A	0.9241	0.2435	0.2285	0.193*	
H1B	0.8229	0.1904	0.0962	0.193*	
H1C	0.7809	0.1795	0.2605	0.193*	

### Atomic displacement parameters (Å^2^)

**Table:**

	*U*^11^	*U*^22^	*U*^33^	*U*^12^	*U*^13^	*U*^23^
N1	0.0380 (10)	0.0516 (11)	0.0319 (9)	0.0030 (9)	0.0030 (7)	0.0023 (8)
N2	0.0407 (11)	0.0834 (16)	0.0336 (10)	0.0013 (10)	0.0097 (8)	0.0031 (10)
C10	0.0364 (11)	0.0564 (15)	0.0413 (12)	0.0045 (11)	0.0017 (9)	0.0019 (10)
O2	0.0728 (13)	0.0805 (15)	0.0564 (12)	0.0151 (11)	−0.0018 (9)	−0.0074 (10)
O1	0.0988 (16)	0.0791 (15)	0.0457 (11)	0.0097 (12)	−0.0087 (10)	0.0001 (9)
C9	0.0377 (11)	0.0553 (14)	0.0304 (11)	0.0037 (11)	0.0033 (8)	−0.0010 (9)
C8	0.0518 (14)	0.0532 (15)	0.0491 (15)	−0.0082 (12)	0.0006 (11)	0.0019 (11)
C7	0.0522 (15)	0.086 (2)	0.0343 (12)	−0.0015 (13)	0.0134 (10)	0.0047 (12)
C6	0.0539 (14)	0.0675 (17)	0.0289 (11)	0.0016 (12)	0.0051 (10)	0.0033 (11)
C5	0.0474 (13)	0.0629 (16)	0.0444 (13)	−0.0046 (12)	0.0054 (10)	0.0044 (12)
C4	0.100 (3)	0.098 (3)	0.069 (2)	0.020 (2)	0.0058 (18)	−0.0199 (19)
C3	0.097 (3)	0.095 (3)	0.098 (3)	−0.005 (2)	0.026 (2)	−0.021 (2)
C2	0.101 (3)	0.095 (3)	0.115 (3)	−0.006 (2)	0.013 (2)	−0.001 (2)
C1	0.087 (3)	0.101 (3)	0.198 (5)	0.000 (2)	0.025 (3)	0.031 (3)

### Geometric parameters (Å, °)

**Table:**

N1—C9	1.371 (3)	C6—H6A	0.9300
N1—C6	1.376 (3)	C5—H5A	0.9700
N1—C10	1.468 (3)	C5—H5B	0.9700
N2—C9	1.331 (3)	C4—C3	1.480 (5)
N2—C7	1.378 (3)	C4—H4A	0.9700
C10—C5	1.516 (3)	C4—H4B	0.9700
C10—H10A	0.9700	C3—C2	1.473 (5)
C10—H10B	0.9700	C3—H3A	0.9700
O2—C8	1.329 (3)	C3—H3B	0.9700
O2—C4	1.475 (4)	C2—C1	1.531 (5)
O1—C8	1.199 (3)	C2—H2B	0.9700
C9—C9^i^	1.460 (4)	C2—H2C	0.9700
C8—C5	1.500 (4)	C1—H1A	0.9600
C7—C6	1.347 (3)	C1—H1B	0.9600
C7—H7A	0.9300	C1—H1C	0.9600
			
C9—N1—C6	106.13 (18)	C10—C5—H5B	109.3
C9—N1—C10	130.15 (19)	H5A—C5—H5B	108.0
C6—N1—C10	123.53 (18)	O2—C4—C3	108.9 (3)
C9—N2—C7	104.6 (2)	O2—C4—H4A	109.9
N1—C10—C5	112.1 (2)	C3—C4—H4A	109.9
N1—C10—H10A	109.2	O2—C4—H4B	109.9
C5—C10—H10A	109.2	C3—C4—H4B	109.9
N1—C10—H10B	109.2	H4A—C4—H4B	108.3
C5—C10—H10B	109.2	C2—C3—C4	115.3 (4)
H10A—C10—H10B	107.9	C2—C3—H3A	108.4
C8—O2—C4	116.1 (2)	C4—C3—H3A	108.4
N2—C9—N1	111.59 (19)	C2—C3—H3B	108.4
N2—C9—C9^i^	124.5 (2)	C4—C3—H3B	108.4
N1—C9—C9^i^	123.9 (2)	H3A—C3—H3B	107.5
O1—C8—O2	122.7 (3)	C3—C2—C1	112.7 (4)
O1—C8—C5	124.7 (3)	C3—C2—H2B	109.0
O2—C8—C5	112.6 (2)	C1—C2—H2B	109.0
C6—C7—N2	110.9 (2)	C3—C2—H2C	109.0
C6—C7—H7A	124.5	C1—C2—H2C	109.0
N2—C7—H7A	124.5	H2B—C2—H2C	107.8
C7—C6—N1	106.7 (2)	C2—C1—H1A	109.5
C7—C6—H6A	126.6	C2—C1—H1B	109.5
N1—C6—H6A	126.6	H1A—C1—H1B	109.5
C8—C5—C10	111.6 (2)	C2—C1—H1C	109.5
C8—C5—H5A	109.3	H1A—C1—H1C	109.5
C10—C5—H5A	109.3	H1B—C1—H1C	109.5
C8—C5—H5B	109.3		

Symmetry codes: (i) −*x*+1, −*y*+1, −*z*+1.

### Hydrogen-bond geometry (Å, °)

**Table:**

*D*—H···*A*	*D*—H	H···*A*	*D*···*A*	*D*—H···*A*
C10—H10B···N2^i^	0.97	2.46	2.960 (3)	111

Symmetry codes: (i) −*x*+1, −*y*+1, −*z*+1.
